# Non-mass lesions on breast ultrasound: why does not the ACR BI-RADS breast ultrasound lexicon add the terminology?

**DOI:** 10.1007/s10396-023-01291-1

**Published:** 2023-03-11

**Authors:** Takayoshi Uematsu

**Affiliations:** Department of Breast Imaging and Breast Intervention Radiology, Department of Clinical Physiology, Shizuoka Cancer Center Hospital, Nagaizumi, Japan

**Keywords:** Breast, Non-mass, Ultrasound, MRI, BI-RADS

## Abstract

The definition of a non-mass lesion on breast ultrasound (US) is designed for everyday practice to provide unambiguous clinical management and to assist physicians and sonographers as they interpret breast US images. The field of breast imaging research requires consistent and standardized terminology for non-mass lesions identified on breast US, especially when differentiating benign from malignant lesions. Physicians and sonographers should be aware of the benefits and limitations of the terminology and use them precisely. I am hopeful that the next edition of the Breast Imaging Reporting and Data System (BI-RADS) lexicon will include standardized terminology for describing non-mass lesions detected on breast US.

## Introduction

Terms describing non-mass lesions on breast ultrasound (US) are widely used and accepted in screening and clinical settings. Ideally, this definition should facilitate the provision of unambiguous clinical management. However, there is an unmet need for consistent and standardized terminology to describe non-mass lesions detected on breast US to assist physicians and sonographers in interpreting US findings. Unlike mammography, US and magnetic resonance imaging (MRI) are tomographic modalities that display three-dimensional (3D) objects unaffected by the presence of adjacent dense fibroglandular tissue. In addition, contrast-enhanced breast MRI is powerful and highly sensitive for detecting breast cancer; however, its specificity is limited. This ambiguity often necessitates biopsy and histological analysis of suspicious MRI-detected lesions for optimal management [1].

Suspicious lesions with non-mass enhancement detected on MRI are frequently undetected on second-look US, with only around half appearing as non-mass lesions [2]. Integrating the US and MRI lexicon is logical and may help streamline clinical management. For example, ductal carcinoma in situ (DCIS) and invasive lobular carcinoma (ILC) usually manifest as non-mass lesions on breast US and MRI [3, 4]. Despite this, the American College of Radiology Breast Imaging Reporting and Data System (ACR BI-RADS) breast US lexicon does not contain this information. This omission may seed confusion about how to describe and manage non-mass lesions detected on breast US. I recommend using this article’s definition of a non-mass lesion on breast US to promote use of a standardized terminology.

## Definition of non-mass lesions on breast US

On breast US, a non-mass lesion is a hypoechoic area that has an indistinct shape on two different projections, but that does not fit the criteria of a mass; that is, it lacks convex outer borders and conspicuity [3]. This definition was used in at least 47 articles. The proposed definition of breast non-mass lesions is based on normal breast anatomy and will inform physicians’ and sonographers’ interpretations of US findings.

**Fig. 1:**
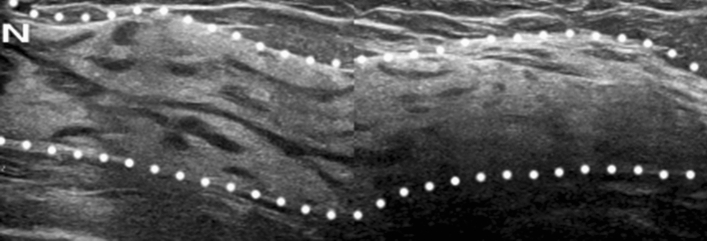
Normal breast tissue depicted on US in a 46-year-old woman. Normal fibroglandular tissue (between the dotted lines) is composed of gray dendritic structures (ducts and lobules with surrounding hard stroma) and a white area (edematous and fat-containing stroma). N indicates the nipple

**Fig. 2:**
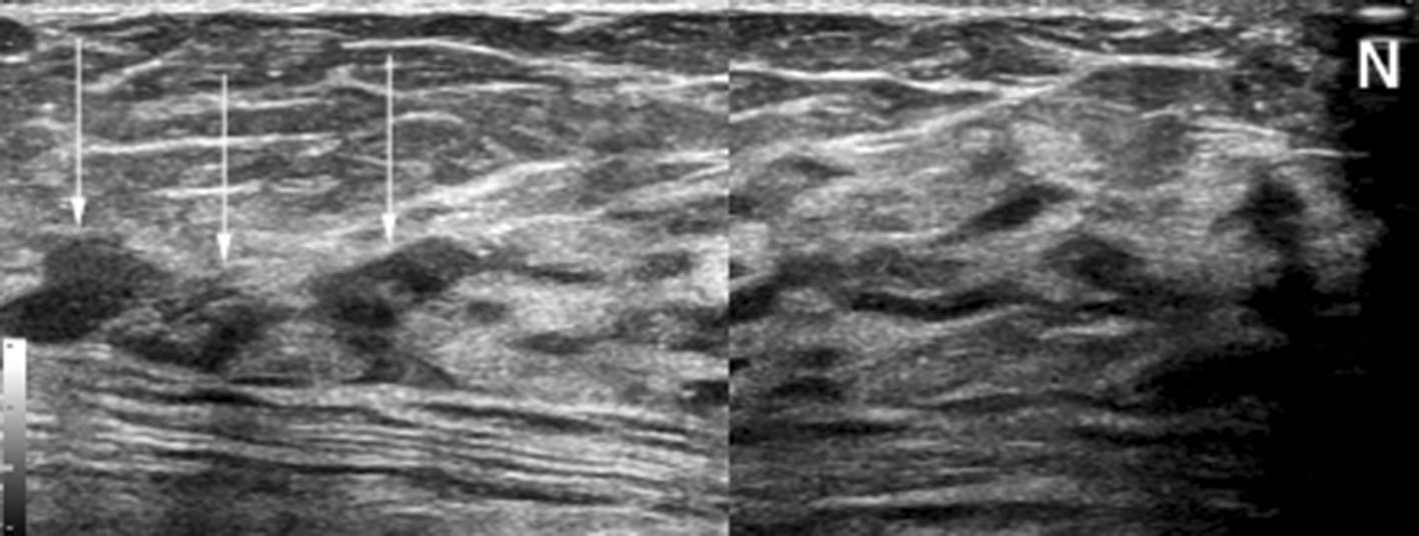
Suspicious duct and lobule patterns on breast US in a 71-year-old woman. This US image shows a non-mass lesion (arrows) at the edge of the breast with disrupted duct tapering. Normally, duct and lobule patterns tapering off at the edge of the breast are noted (see Fig. 1). The results of the pathological examination confirmed LCIS. N indicates the nipple

Breast US can identify ducts and lobules, with surrounding hard stroma depicted in gray. US also depicts fibrous tissue, including edematous and fat-containing stroma, as a white area in the breast (Fig. 1) [5]. Fibroglandular tissue is depicted as a mixture of gray and white (Fig. 1). The gray dendritic structures present normal ducts and lobules on breast US (Fig. 1). On breast US, non-mass lesions will feature abnormal duct and lobule patterns (Fig. 2).

Importantly, DCIS and ILC frequently manifest as non-mass lesions on breast US [3, 4] and feature focal discontinuities and disruptions in the gray dendritic structures that otherwise characterize normal ducts and lobules. Unfortunately, the classification scheme for non-mass image-forming findings on breast US proposed by the Japan Association of Breast and Thyroid Sonology since 2004 [6] is unnecessarily complicated. This classification scheme differs from the simple and practical definition of non-mass lesions on breast US offered here.

## Associated findings of non-mass lesions on breast US

### Calcifications

**Fig. 3:**
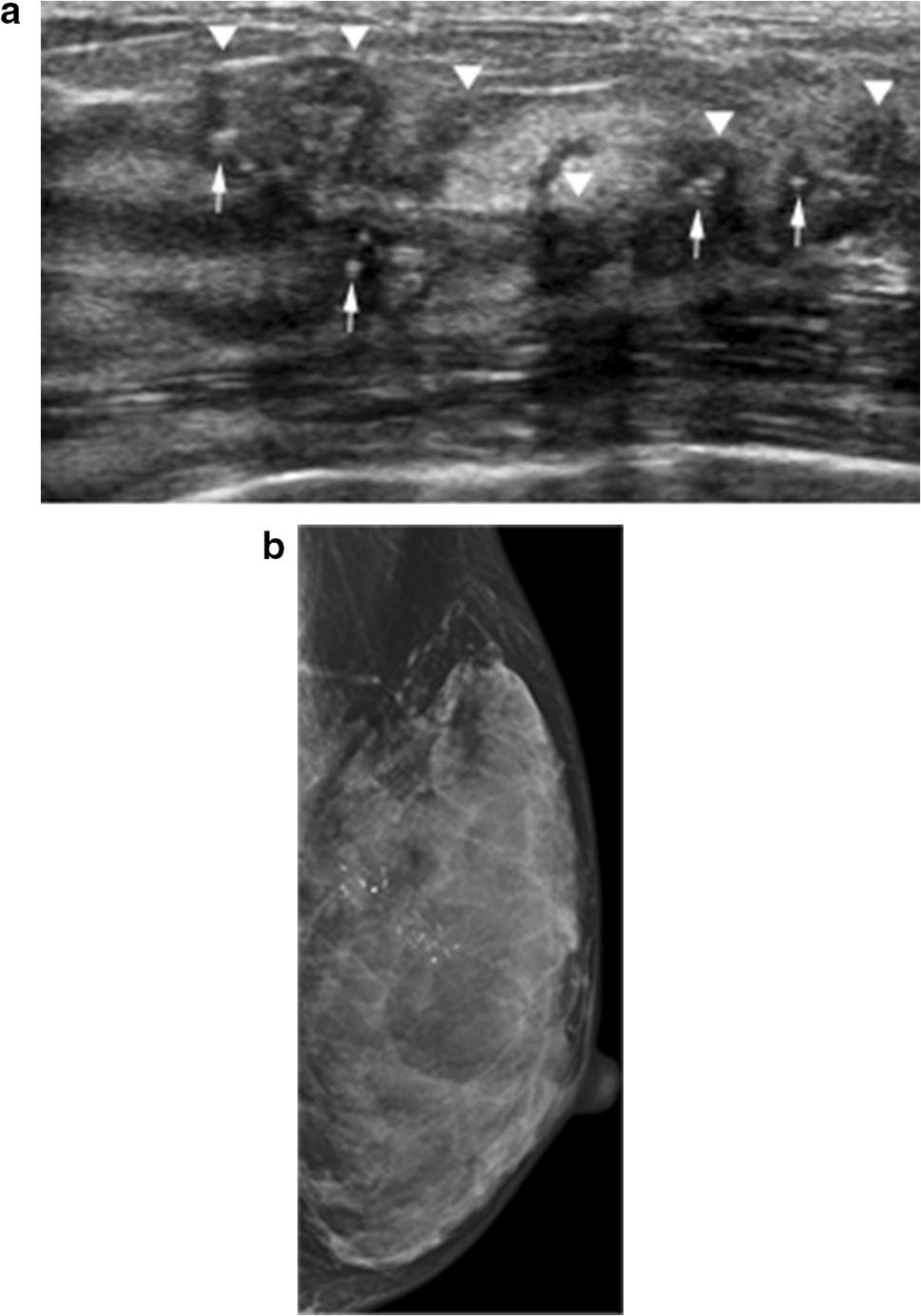
Non-mass lesions with associated calcifications on breast US in a 61-year-old woman. **a** The US image shows a non-mass lesion (arrowheads) with internal hypoechoic echogenicity and small hyperechoic foci (arrows). **b** The mammogram shows segmental fine pleomorphic and line-branching calcifications. A subsequent pathological examination confirmed high-grade DCIS

Non-mass lesions often include echo patterns that extend over hypoechoic or heterogeneous echogenicity. Internal echoic characteristics with small hyperechoic foci (i.e., calcifications) differ from surrounding normal gray dendritic structures, which depict normal ducts and lobules. Therefore, non-mass lesions with associated calcifications on breast US (Fig. 3) tend to be malignant [7–10] and should be classified as suspicious and biopsied.

### Architectural distortion

**Fig. 4:**
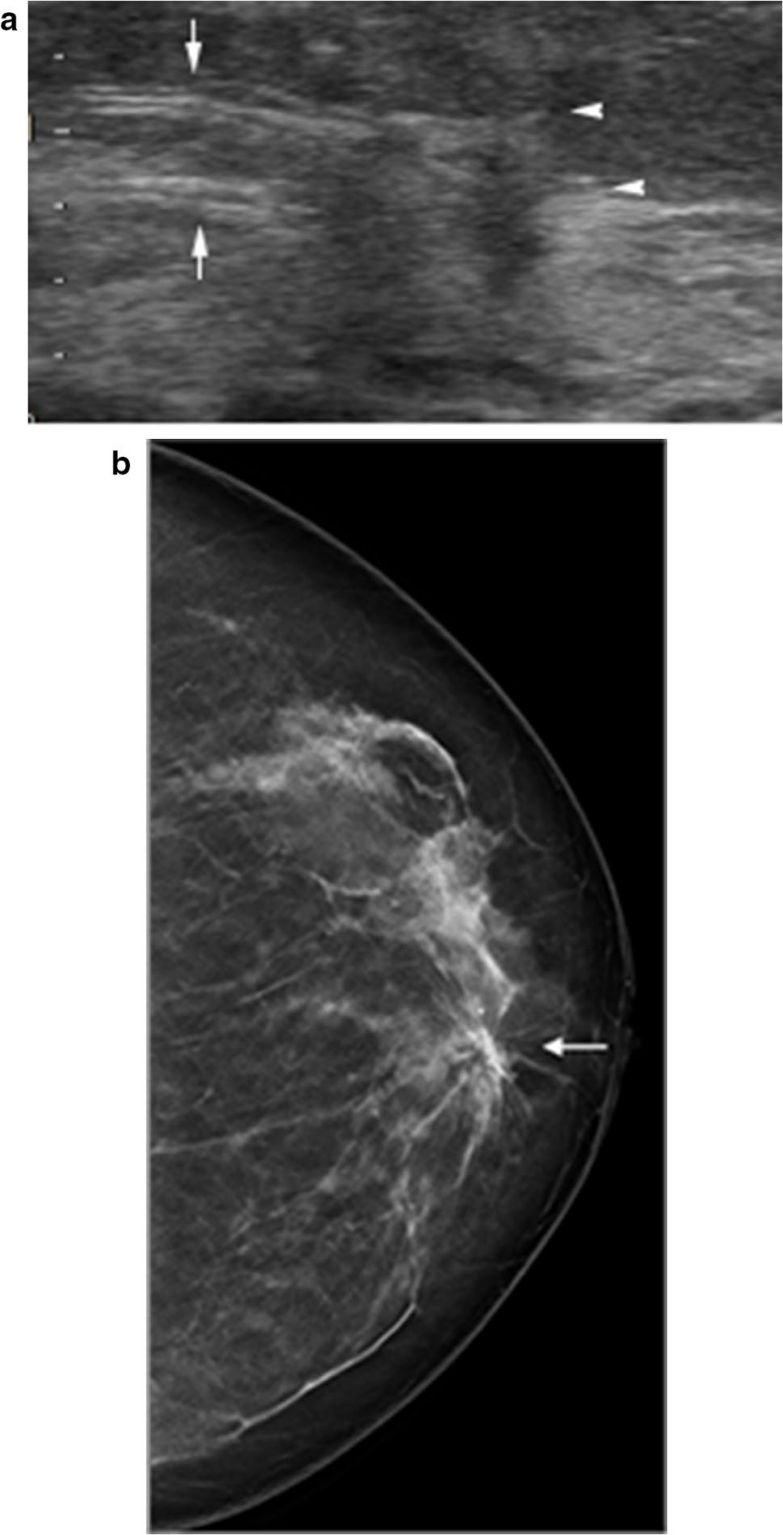
Non-mass lesions with associated architectural distortion on breast US in a 70-year-old woman. **a** The US image shows a non-mass lesion with thin straight lines (arrows) and radiating spiculations (arrowheads). **b** The breast tomosynthesis image shows spiculations (arrow). A subsequent pathological examination confirmed sclerosing adenosis

Architectural distortion on breast US means that fibroglandular tissue is distorted by non-mass lesions. This may include thin straight lines, radiating spiculations from non-masses, and focal retraction at the fibroglandular tissue's anterior or posterior edge. While non-mass lesions with associated architectural distortion on breast US tend to be malignant [9, 10], they can be benign lesions (Fig. 4). These findings should be classified as suspicious and biopsied.

### High elasticity

**Fig. 5:**
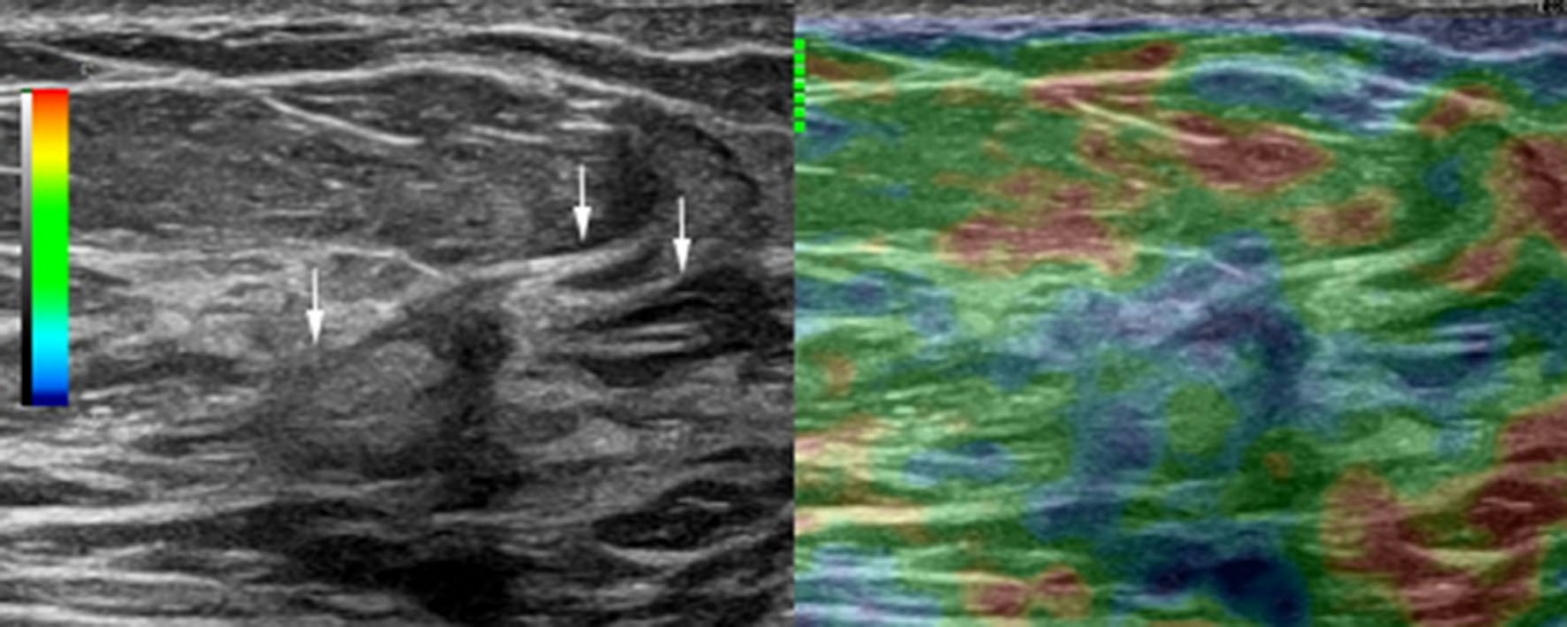
US strain and B-mode images in split-screen mode in a 70-year-old woman. The right B-mode image shows a non-mass lesion with radiating spiculations (arrows). The left real-time elastography image depicts the lesion in blue, indicating high strain and an elasticity score of 4. A subsequent pathological examination confirmed ILC

Shear wave elastography quantifies a tissue's elasticity in kilopascals or meters per second, and the color-coded images are generated in real time by local estimation of shear wave propagation speed. This method improves the diagnostic specificity of breast US for non-mass lesions [9]. Real-time elastography can assist in diagnosis, given its increased specificity for distinguishing benign from malignant non-mass lesions (Fig. 5).

### Hypervascularity

**Fig. 6:**
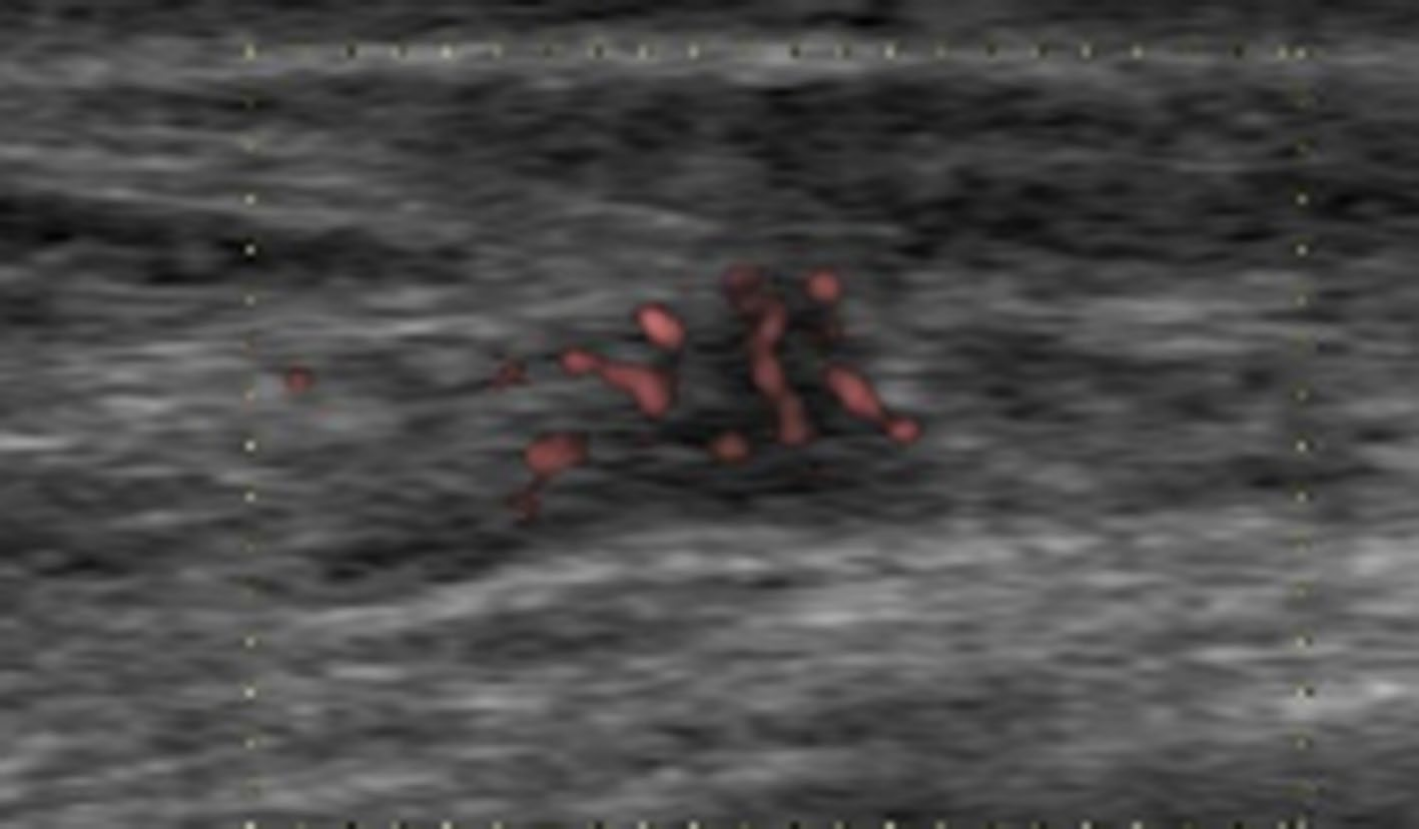
The power Doppler US image shows a non-mass lesion with some internal vascularity in a 76-year-old woman. A subsequent pathological examination confirmed intermediate-grade DCIS

Color or power Doppler US is used to depict blood vessels in non-mass lesions. Malignant non-mass lesions feature significantly higher vascularity (more than two vessels) than benign non-mass lesions (Fig. 6). Color Doppler US improves the specificity of breast US for characterizing non-mass lesions [9]. Elasticity and vascularity results can help characterize non-mass lesions on breast US better than morphological features on B-mode US.

## Correlation between MRI and US depictions of non-mass lesions

Contrast-enhanced breast MRI is a powerful and highly sensitive breast imaging tool; however, its specificity is limited [1]. Suspicious MRI-detected lesions require a biopsy and histological examination to determine the optimal management. Suspicious MRI-detected lesions with non-mass enhancement are frequently undetected on second-look US. Approximately half of the malignant lesions with non-mass enhancement on MRI appeared as non-mass lesions on second-look US [2]. MRI-detected lesions can appear so subtle on breast US that they tend to be classified as non-mass lesions [1, 11, 12]. The ACR BI-RADS breast US lexicon inadequately describes such lesions, and their management and follow-up still need to be standardized.

DCIS is often diagnosed as non-mass enhancement on MRI [13]. Non-mass findings on breast US should be considered analogous to non-mass findings according to the ACR BI-RADS breast MRI lexicon [3]. Wherever possible and appropriate, we should use standardized terminology to describe non-mass lesions on both breast MRI and US.

## Non-mass enhancement distribution

**Fig. 7:**
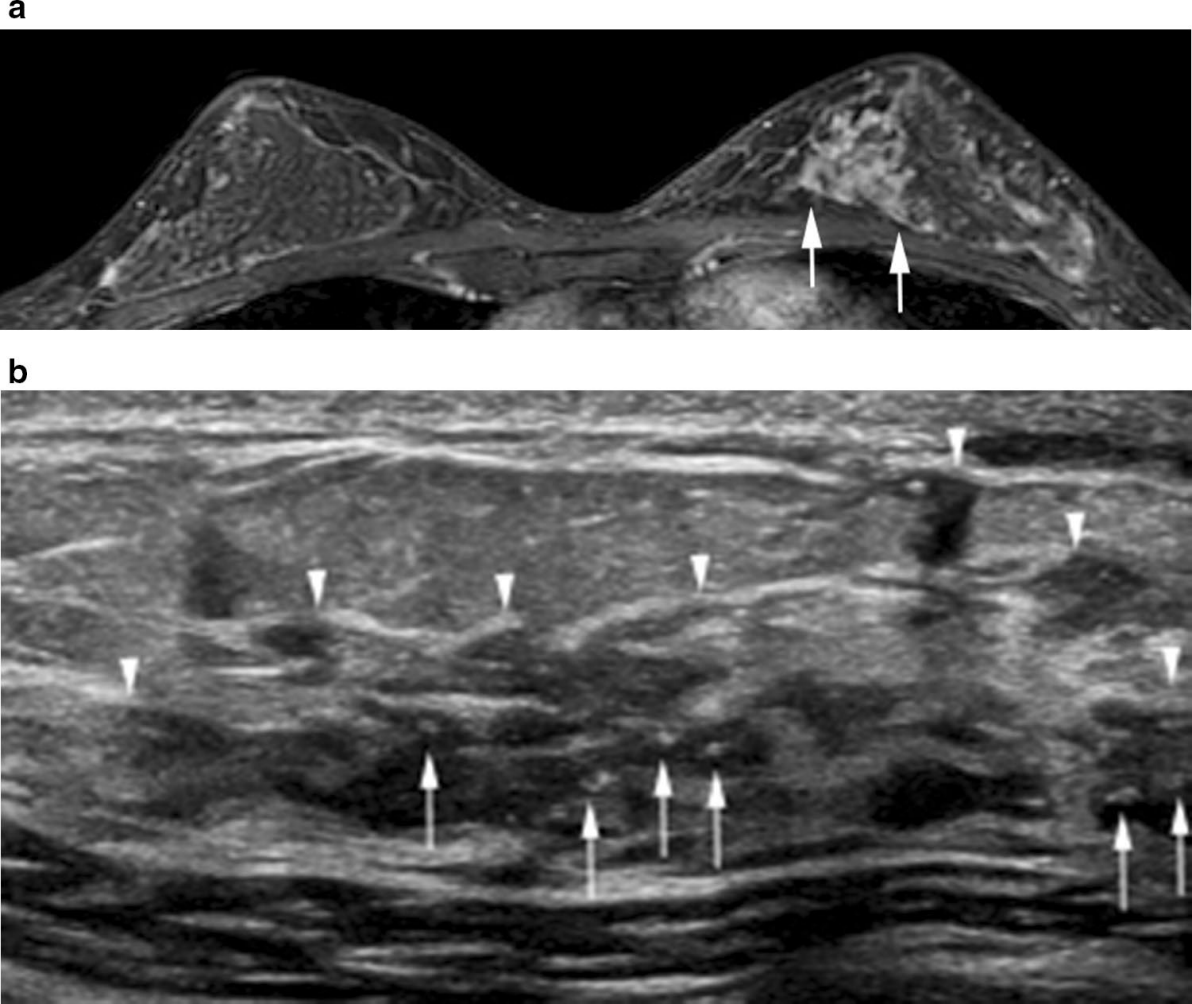
High-grade DCIS in the left upper inner breast in a 43-year-old woman. **a** The axial postcontrast T1-weighted image shows segmental enhancement (arrows). **b** The corresponding US image shows a segmental non-mass lesion (arrowheads) with associated calcifications (arrows)

**Fig. 8:**
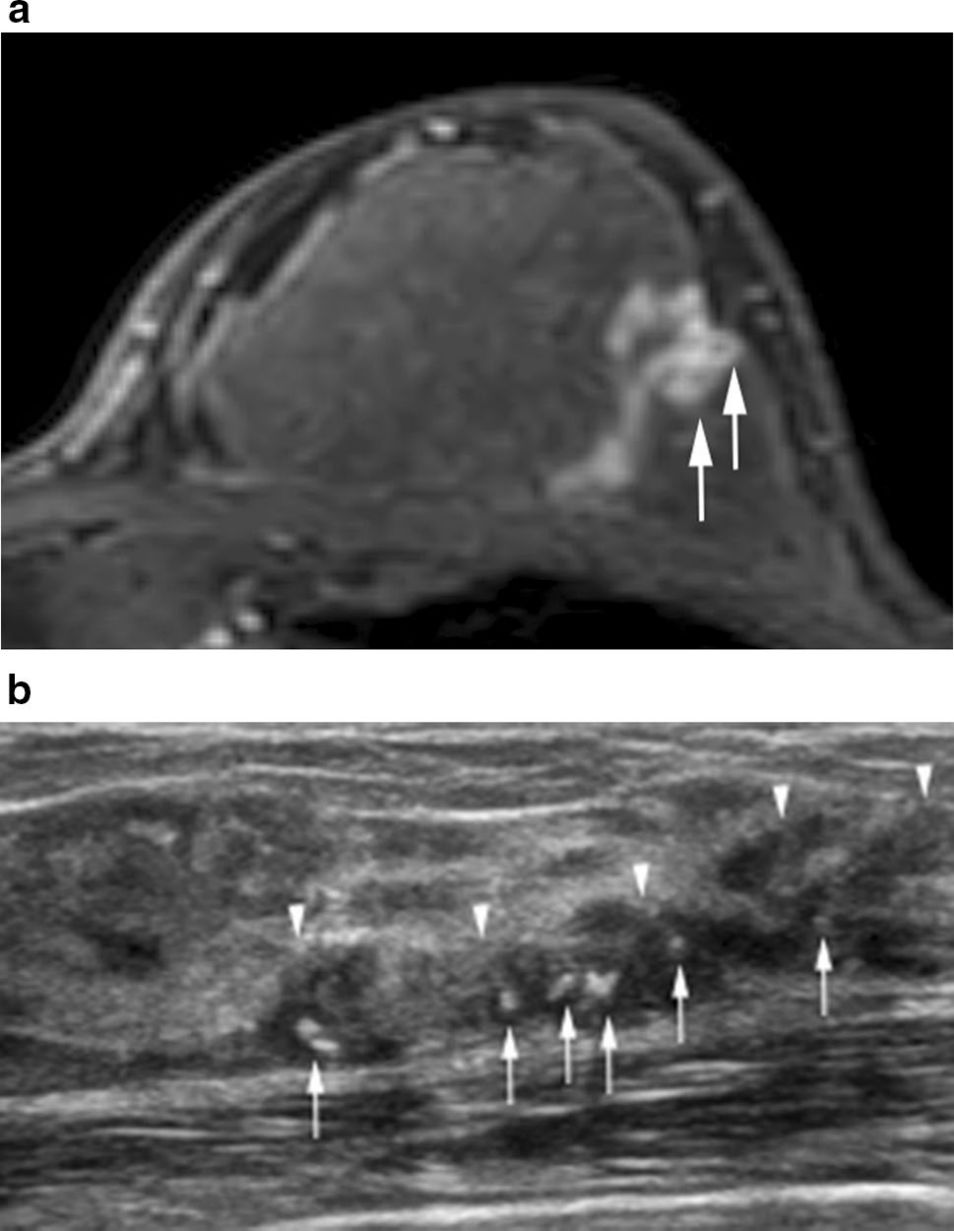
High-grade DCIS in the left upper outer breast in a 61-year-old woman. **a** The axial postcontrast T1-weighted image shows clumped linear enhancement with clustered ring enhancements (arrows). **b** The corresponding US image shows a clumped linear non-mass lesion (arrowheads) with associated calcifications (arrows)

There are several different non-mass enhancement distribution patterns on breast MRI. Segmental or linear enhancement patterns are typical of DCIS on MRI; the corresponding images on breast US are usually in agreement with MRI findings (Figs. 7 and 8) [13].

## Non-mass internal enhancement pattern

DCIS is characterized by various non-mass enhancements of internal patterns on breast MRI; however, clumped or clustered patterns are the most common (Fig. 8) [13].

## Conclusion

Understanding the clinical relevance of non-mass findings on breast US is imperative for managing a patient's diagnosis. Categorizing hypoechoic areas with indistinct boundaries as “mass” or “non-mass” lesions can be problematic. A mass lesion with indistinct margins may appear as a non-mass lesion to other readers. Physicians and sonographers should be aware of the benefits and limitations of finding classification schemes. Because we have no standardized definition of “non-mass” lesions included in the ACR BI-RADS, confusion about how to describe and manage these lesions is expected. I am hopeful that the new BI-RADS breast US lexicon will include standardized terminology for describing non-mass lesions on breast US.

## Data Availability

The author has no relationships relevant to the contents of this paper to disclose.
